# The Influence of Care Home Managers' Leadership on the Delivery of Person-Centred Care for People Living with Dementia: A Systematic Review

**DOI:** 10.1155/2023/9872272

**Published:** 2023-05-17

**Authors:** Linda Moenke, Melanie Handley, Claire Goodman

**Affiliations:** ^1^Centre for Research in Public Health and Community Care (CRIPACC), University of Hertfordshire, Hatfield, UK; ^2^National Institute for Health Research (NIHR), Applied Research Collaboration (ARC) East of England, Cambridgeshire, UK

## Abstract

**Background:**

Care home managers' leadership is recognised as directly influencing the care received by people living with dementia. What enables care home managers to promote and sustain person-centred care for residents is less well understood.

**Method:**

A mixed-methods systematic review synthesised evidence on care home managers' leadership on the delivery of person-centred care for people living with dementia. Electronic databases (PubMed, Scopus, Cochrane Library, CINAHL, and Google Scholar) were searched between 2009-2021. Thematic synthesis identified commonalities, facilitators, and barriers to managers enabling person-centred care.

**Results:**

Twenty-one studies met the inclusion criteria. Approaches demonstrated by care home managers that enabled person-centred care for people living with dementia included valuing and recognising staffs' work; involving residents and relatives in decision making; providing feedback to staff; promoting a positive work environment and care culture; and involving staff in organisational changes. Barriers to person-centred care were a lack of organisational support for care home managers; staff shortages; managers not having time to work with staff; manager-staff turnover; limited access to dementia training; and a lack of leadership education and training for care home managers.

**Conclusion:**

Care home managers are central to the delivery of person-centred care for people living with dementia. The review identified key resources and activities that support this work. The wide variation in leadership approach and a persistent lack of detail about the frequency of educational and organisational support demonstrate a need to explore what enables care home managers to support their staff to deliver person-centred care.

## 1. Introduction

Care homes play a vital role in providing care for older people living with complex health conditions and care needs. In the UK, there are 420,000 care home residents, 80% of whom are living with dementia or cognitive impairment [1]. For this population, person-centred care is integral. There is not a single definition of person-centred care, but it describes an approach to care where people living with dementia are not defined by their condition [2]. This assumes that the person living with dementia is involved in decision making about their care and that their personal history, preferences, and priorities directly inform their care [3, 4]. Person-centred interventions can enhance the psychosocial care environment and encourage social stimulation and interaction for people living with dementia [5, 6]. To achieve this requires care home managers enabling staff to meet the individual needs of people living with dementia [7]. While guidance and theoretical frameworks set out person-centred approaches, less is clear about what care home managers need to sustain the practice [8, 9].

Care home managers are central to the effectiveness of care home services, but their work has often been overlooked in research [10]. Leadership influences not only the retention of skilled staff [11, 12], but also the quality of care that is being delivered to care home residents [13]. Studies report leadership as “impactful” to person-centred care for people living with dementia in care homes [14–16]. However, the kind of support required to improve the delivery of person-centred care for people living with dementia lacks detail [17, 18].

Studies that have explored how to improve care home leadership argue that training and education are essential for person-centred care practices [19–23]. Supporting person-centred care requires care home staff to develop a complex set of qualities or skills which go beyond providing personal care [24]. Brooker [25] argues that care homes with a person-centred culture “value” the rights of the person living with dementia, provide “individualised” care based on the person's “perspective” of what matters and meet the person's “social” environmental needs. How care home managers' leadership directly influences person-centred care delivery is the focus of this review.

### 1.1. Aims and Objectives

The aim of the review was to understand how care home managers enable the delivery of person-centred care for people living with dementia. The objectives were:

1. To review the evidence on how care home managers' leadership influences the delivery of care for people living with dementia or cognitive impairment;
2. To understand how different leadership strategies or styles support care staffs' roles and responsibilities to provide person-centred care; and
3. To describe what care home managers need to be effective leaders when enabling staff to deliver person-centred care for people living with dementia.

## 2. Methods

We conducted a mixed-methods review [26] to synthesise quantitative and qualitative evidence on diverse aspects of care home leadership and how leadership influenced the delivery of person-centred care. This review followed the Preferred Reporting Items for Systematic Reviews and Meta-Analysis (PRISMA) guidelines and checklist [27]. The review protocol was registered on PROSPERO (CRD42021237930).

### 2.1. Search Strategy

A systematic search of five databases (PubMed, Scopus, Cochrane Library, CINAHL, and Google Scholar) was completed for research published between 2009-2021. The start date reflects changes in care home regulation in England which included the assessment of care home leadership by the Care Quality Commission (CQC) (1^st^ April 2009). Forward and backward citation searches were completed for included studies and excluded reviews. Examples of electronic databases searched and terms used are given in Table 1.

### 2.2. Inclusion and Exclusion Criteria

All types of primary research were eligible for inclusion. Studies were included if they were published in English language and reported on leadership in care homes (with and without onsite nursing) for people living with dementia or older people with cognitive impairment in countries with similar healthcare systems to the UK. The similarity of healthcare systems and for this paper long-term care was decided on the basis that the two systems were linked but separately funded and organised [28]. Studies that focused on palliative care for people living with dementia in care homes were excluded due to the specific responsibilities and requirements of such care.

### 2.3. Screening

Results of electronic searches were downloaded into bibliographic software, and duplicates were removed. One reviewer (LM) screened titles and abstracts for eligibility against the inclusion criteria, with 20% screened by a second reviewer (MH). All full-texts of potentially eligible studies were reviewed by LM, with 20% double-screened by MH. Disagreements were resolved through discussion with all reviewers (LM, MH, and CG).

### 2.4. Data Extraction

A bespoke data extraction form was developed for use across all studies. One reviewer (LM) extracted data from all studies, with 10% checked for accuracy by a second reviewer (MH). Data were extracted on publication characteristics, study design, setting, data collection method, participant characteristics, study outcomes, leadership styles, and organisational culture.

### 2.5. Synthesis

Narrative synthesis was used to summarise findings across studies [29]. The heterogeneity of research designs and diversity in outcome measures meant meta-analysis was not feasible [30]. Findings were tabulated and presented as descriptive accounts. The synthesis then followed thematic analysis [31] to familiarise data, generate codes, and identify themes. Each code described the statements and responses reported in the included studies. Patterns were identified within and across the codes, generating three analytical themes.

### 2.6. Appraisal of the Included Studies

Studies were assessed by one reviewer (LM) using appraisal tools appropriate to the study design to assess the risk of bias in the included studies. Tools used were as follows: Appraisal tool for Cross-sectional Studies (AXIS) for quantitative studies [32]; Critical Appraisal Skill Programme (CASP) for qualitative studies [33], and Mixed Methods Appraisal Tool (MMAT) for mixed-methods studies [34]. Details of the appraisal tools and scoring criteria used to assess studies for risk of bias are provided in the full version (Supporting Information 1).

## 3. Findings

**Figure 1:**
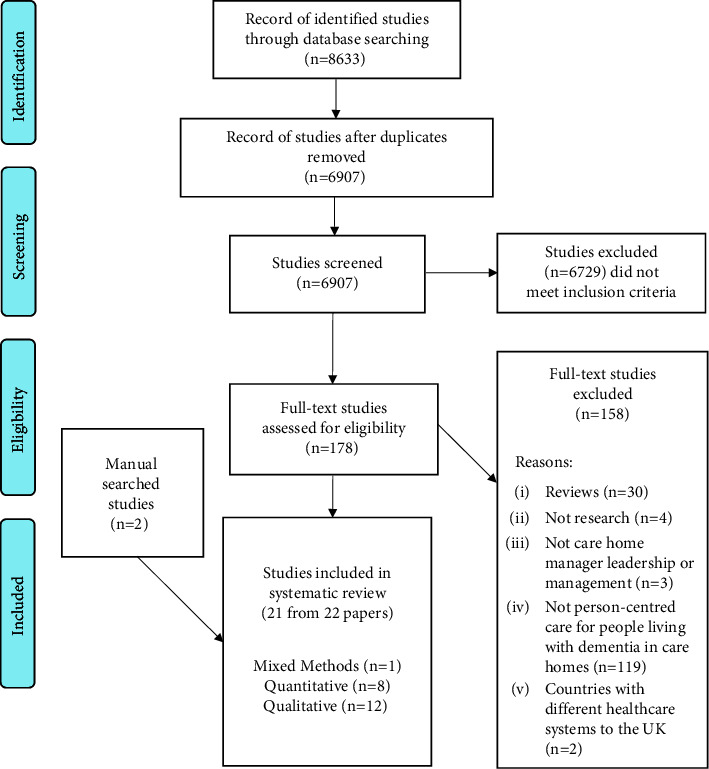
PRISMA flowchart illustrating the search strategy and study selection process.

Searches identified 8,633 potentially relevant documents. After deduplication, 6,907 were screened by title and abstract, with 178 records taken forward to the full-text screening. A total of 158 records were excluded as follows reviews (*n* = 30); not research (*n* = 4); not related to care home managers' leadership (*n* = 3); not related to person-centred care for people living with dementia in care homes (*n* = 119); and countries with different healthcare systems to the UK (*n* = 2). The two studies that were excluded originated from South Africa and China. The decision to exclude the studies was on the basis that the systems of care were immensely different and the findings did not offer transferable learning. Two additional papers were identified by manual searches, resulting in 22 included papers reporting findings from 21 studies. The search strategy and study selection process are illustrated in the PRISMA flowchart [27] (Figure 1).

### 3.1. Quality Assessment

Overall, 55% of the included studies scored low and 45% scored medium for risks of bias.

### 3.2. Characteristics of the Included Studies

The study designs were qualitative (*n* = 12), quantitative (*n* = 8), and mixed-methods (*n* = 1). Data collection methods used were observations and interviews (*n* = 2), focus groups (*n* = 5), interviews (*n* = 5), and questionnaires (*n* = 9). Six studies were conducted in the UK [35–41], five in Norway [42–46], three in Sweden [47–49], two in Australia [50, 51], two in the Netherlands [52, 53], one in the USA [54], one in Canada [55], and one in Germany [56]. Studies in care home settings identified as nursing homes, residential aged care facilities, and long-term care facilities. The participant groups in the studies were care home assistants [38, 43, 45–48, 53, 55], care home managers [36, 37, 40, 51, 56], combination of care home assistants and managers [39, 41, 44, 49], and care home assistants and residents [52]. Three studies included a wider mix of participants including directors, managers, care home assistants and relatives (*n* = 2) [42, 54], managers, relatives and care home assistants [35], managers, care home assistants, and nondirect care staff [50]. An overview of twenty-one included studies' characteristics, primary focus and assessment tools and scores are provided in Table 2.

**Figure 2:**
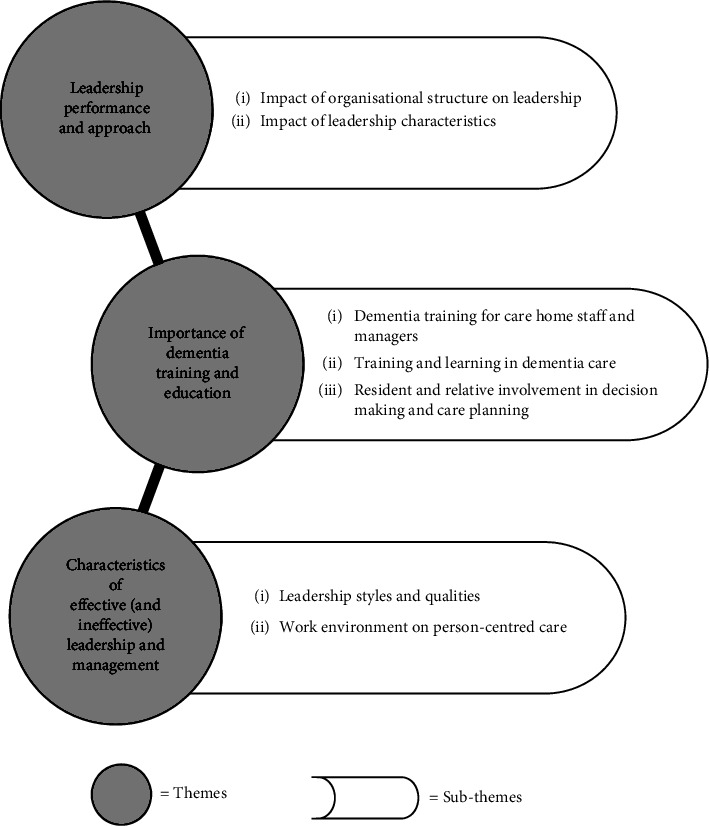
Three themes and nine subthemes from the results of the review.

Three main themes were identified on how care home managers influence the delivery of person-centred care for people living with dementia: (1) leadership performance and approach; (2) impact of dementia care education and training; and (3) characteristics of effective (and ineffective) leadership and management (Table 3, Figure 2).

### 3.3. Theme 1: Leadership Performance and Approach

This theme encompasses what type of leadership approach enables staff to implement person-centred practices. It highlights how different care home organisational structures affect managers' ability to enable person-centred care for people living with dementia.

#### 3.3.1. Impact of Organisational Structure on Leadership

The ownership and organisational structure of a care homes affected how care home managers were able to prioritise person-centred approaches to residents' care. Policies and procedures that supported work-life balance (i.e., human resources recognition of staffs' commitments outside the care home and manageable shift patterns) were described as key to the delivery of person-centred care [55]. The majority of papers reviewed, however, identified how policies and procedures that did not prioritise residents' care needs affected the ways that staff were supported to deliver person-centred care ([37, 38, 40, 41, 45, 50, 51, 55, 56]).

When care home managers were visible and regularly affirmed staffs' approach to residents' care, this was reported as improving staff satisfaction and motivation to personalise residents' care [47]. Excess paperwork and administrative duties limited care home managers' capacity to engage with staff and reinforce a good care practice [36, 37]. Changes in care home ownership and management, structural reorganisations, and staff turnover destabilised leadership and caused uncertainties and staff frustration around providing person-centred care for people living with dementia [38, 40, 41].

#### 3.3.2. Impact of Leadership Characteristics

Four studies identified that staff relied on care home managers being affirming and supportive [35, 47, 52, 55]. Where this did not happen, staff reported feelings of low self-esteem and a lack of confidence in their role. Low staff morale, absence at work, and stress compounded reported difficulties in providing person-centred care to people living with dementia [45, 47, 48, 50, 55]. Two UK studies evaluated care home managers implementing a mindful leadership approach [36, 37]. The My Home Life (MHL) leadership programme focused on enabling managers to create extra time outside their administrative duties to develop relationships with staff and work closely as a team. This was observed to be essential for improving person-centred care [36, 37]. As part of this process, care home managers used regular staff supervisions to address specific instances of ageism and depersonalised language towards people living with dementia. For example, prior to the changes, staff were observed using inappropriate language such as the term “feeders” to describe residents that required assistance with feeding and “doubles” for residents that required assistance with mobility by two staff members. The MHL leadership support programme enabled care home managers to reflect closely on their day-to-day responsibility and recognise what practices they needed to change to develop their leadership skills [36, 37]. This approach of listening to staff, involving them in organisational changes, and maintaining relationships and interaction with residents and relatives was found to improve staff morale and productivity to improve care quality [36, 37, 51]. Where these skills were lacking due to an absence of teamwork and limited regular and reliable support from care home managers, staffs' confidence to provide good care to residents could be affected [51].

### 3.4. Theme 2: Importance of Dementia Training and Education

This theme explains how the knowledge acquired through dementia care training and education influences the way person-centred care is delivered to people living with dementia in care homes.

#### 3.4.1. Dementia Training for Care Home Staff and Managers

Where there was access to dementia training for staff and care home managers, this informed the leadership work of care home managers and affected staff confidence and motivation to provide care to residents with dignity and empathy [39, 43, 48]. In two Scandinavian studies, the delivery of person-centred care was positively associated with national leadership education for care home managers [49], and health-related continuing education of three years or more for staff [45]. Shared training involving both staff and managers built a common understanding of care priorities and person-centred care home culture. This quote summarises how staff reported what was important in how they provided care. It also illustrates the tensions of being person-centred, managing risk, and caring in communal environments:

“*The residents are to be allowed to decide by themselves, as long as their own or other people's security or health are not under threat*.*”* [43].

Care home managers who attended dementia training reported how they adopted new activities and psychosocial approaches into residents' care. The activities included supporting staff to deliver one-to-one sessions, hand massages, cookery classes, group dance, and music sessions [39]. Practical solutions and examples of care included in the training appeared to be particularly valued. One care home manager observed how person-centred care, when done well, could reduce the use of antipsychotic medication:

Dementia training improved staffs' communication skills such as keeping language simple, asking short questions, and using pictorial communication methods. Despite positive staff feedback on how their care practice changed, minimal improvements were observed in one study on residents' agitation and well-being following dementia training. This increased opportunities for interactive learning activities for staff; however, they were not sufficient to achieve measurable change [39]. It raised care home managers' awareness about address how information about residents was used to provide person-centred care. For example, making sure that staff incorporate residents' biographical information to maintain individualised care plans [50]. These changes were constrained by residents' life histories not being known or becoming routinised in their day-to-day care [47, 50]. How dementia training was delivered affected learning and uptake. Face-to-face dementia training was more appropriate than handouts, but removed staff from providing care. It was an ongoing challenge for care home managers to assign staff for dementia training despite staff shortages [39].

#### 3.4.2. Training and Learning in Dementia Care

Three studies investigated the use of Dementia Care Mapping (DCM) for improving person-centred care [41, 44, 56]. Dementia Care Mapping, a psychosocial intervention that looks at the frequency and quality of staff-resident interactions, assesses care through observations that are reviewed with staff to inform practice and care planning [41, 44, 56]. In these studies, care home managers trained as “mappers” used DCM to encourage staff to see things from their residents' perspective. Leadership was a key factor in supporting DCM implementation. It required sufficient staff on duty and the allocation of extra time for staff to prioritise activities that deliver person-centred care. Conducting regular staff supervisions was also important for continuity of DCM implementation in care homes [40, 41, 44, 56]. Staff described a positive experience with DCM implementation (i.e., improved communication skills, and increased knowledge of person-centred care) when managers were involved. There were, however, unintended consequences if care home managers were only sporadically available to supervise and support staff or did not understand beforehand the purpose and commitment required. A care home manager explained:

Furthermore, some care home managers misused the DCM to identify the poor job performance of their staff, undermining its purpose [56]. When managers understood the importance of their participation in DCM activities and engaged in the intervention with staff, this motivated them to participate in care activities, and consciously acting as role models [40]. This reinforced teamwork and created opportunities for care home managers to provide constructive feedback to staff [41, 56]. The interventions were not cost neutral, and the need for more staffing resources following DCM intervention became evident as staff became more motivated, confident, and fulfilled in their roles. Successful DCM implementation meant that staff were spending more time with residents. Staff shortages could result in conflicts between care home managers and staff due to staffs' frequent remarks about feeling demoralised and overstretched, and unable to provide person-centred care for people living with dementia [56].

#### 3.4.3. Residents and Relatives Involvement in Decision Making and Care Planning

To embed person-centred care values, people living with dementia should be involved in decision making regarding their care needs in ways that are dementia sensitive [35, 43, 52]. One study identified that for care home managers to enable decision making for people living with dementia, they needed to carry out regular staff observations, resolve daily issues, conduct regular staff meetings, and provide staff supervisions [35]. This increased residents' self-esteem and satisfaction, and reduced agitation. Interactions which facilitate person-centred care between staff and residents also improved shared decision making. If care home managers' participation in residents' care routine was minimal, this could result in little to no involvement of residents and relatives in decision making, and ignoring the use of person-centred care [35, 52]. When staff imposed their own ideas on the residents' wishes and preferences, they were observed to not involve residents in decision making regarding their care [43]. Care home managers who optimised person-centred care were involved in residents' routines, and proactive in encouraging relatives to share information about residents and identify their care needs and preferences. This was particularly important for residents with limited capacity [43, 52].

### 3.5. Theme 3: Characteristics of Effective (and Ineffective) Leadership and Management

This theme describes how the managers' leadership styles which are associated with a supportive work environment for staff and the use of person-centred care.

#### 3.5.1. Leadership Styles and Qualities

A transformational leadership style characterised by how care home managers communicated a vision for improvement, developed staffs' competence, provided support, empowered and motivated staff, led by example, and showed understanding was a predictor of person-centred care [46]. Four studies in the review collected data on how care home managers worked with staff and identified a transformational leadership style as key to enabling the use of person-centred care [46, 52–54]. The ability to solve problems, handle conflicts in a positive way, empower staff, show compassion, and create a positive team were identified as qualities that care home managers drew on to sustain person-centred care [37, 40, 42, 46, 49, 52, 54–56].

A transactional leadership approach which relies on rewards to achieve a goal was considered ineffective for person-centred care delivery [54]. Similarly, care home managers who adopted an authoritative leadership approach (i.e., imposing instructions and top-down decision making) were associated with managers being rarely involved in solving everyday care issues or working alongside staff. This adversely affected how person-centred care was achieved for people living with dementia in care homes [56]. In one study, participants (staff and relatives) reported task-oriented leadership as leading to positive outcomes for staff levels and care quality [42]. Although this was not supported in most of the papers reviewed.

#### 3.5.2. Work Environment for Person-Centred Care

The care home manager influences the organisation of the care home environment. A supportive psychosocial environment characterised by low job strain and increased job satisfaction was associated with higher levels of person-centred care [48]. In contrast to Sjogren et al. [48], other studies described how “high” demands and “low” control in care homes were associated with negative psychosocial environments, leading to stress and lower staff satisfaction [52, 53]. Rutten et al. [53] found that work environment characteristics (i.e., unity between staff, residents and relatives; teamwork and three job characteristics [social support from managers; job satisfaction; and varied roles and development opportunities]) are associated with staff-reported person-centred care. These findings are consistent with previous findings in Ericson-Lidman et al. [47]. Based on this evidence, how care home managers promote a positive work environment (i.e., low job strain and demands, and feelings of job satisfaction for staff) seems to play a crucial role in the use of person-centred care for people living with dementia.

## 4. Discussion

The review asked what kind of leadership is likely to facilitate person-centred care for people living with dementia in care homes. Twenty-one studies were included with six from the UK. Most of the studies were observational with seven studies reporting interventions to promote the delivery of person-centred care. The findings emphasised that how person-centred care is provided to people living with dementia is related to care home managers' leadership approach, dementia care knowledge, involvement in staffs' work and residents' care, staffing levels, and opportunities for manager-staff collaboration. Evidence on how care home managers could be supported was limited to two studies [36, 37]. The literature focused on what care home managers needed to do, but not how care home managers could be supported to improve their leadership skills. None of the studies reviewed explored how care home managers had developed their leadership styles, views about enabling person-centred care, and the impact of prior experiences and educational background. The absence of targeted support for care home managers is likely to be linked to difficulties in maintaining residents' needs [10, 57, 58]. The evidence strongly asserts links between the behaviours of effective managers and the delivery of person-centred care. Less clear was the kind of support and resources that care home managers drew on to enable person-centred care. The association between job satisfaction and person-centred care was only linked to staff and not care home managers. This was despite all the aspects of how care home managers worked being documented as influential in the way that staff provided care to people living with dementia [45, 48, 53].

Care home managers' job satisfaction may be as important as that of their staff. Determinants of care home managers' job satisfaction are: working with constructive and collaborative senior management, having decisional authority, and being provided with resources that support care delivery, such as appropriate IT systems [59]. Future research is needed to explore these different aspects of job satisfaction and preparation that may sustain care home managers' leadership capacity to promote person-centred care for people living with dementia. The review findings supported intervention programmes aimed at assisting care home managers and staff to work together to deliver person-centred dementia care. However, it is unknown whether the frequency of the programme affects and sustains outcomes on care quality. Only one study in the review explored the positive relationship between care home managers' education and person-centred care [49]. The context-sensitive nature of care home managers' work and the multiple pressures on how they work identified in this review also suggest that any leadership education or training programmes should be co-designed, co-produced, and possibly provided in-house to optimise learning and uptake.

Care home managers in the studies reviewed often struggled to maintain care quality for people living with dementia because of workloads, limiting their participation in residents' care within care homes. This resonates with findings from Haunch et al.'s [60] realist review of care home managers' experience of balancing responsibilities across the stakeholders (i.e., residents, relatives, staff, the organisation, the regulator). Future research on providing person-centred care for people living with dementia in care homes could focus on how care home managers' experiences of enabling staff to use person-centred approaches are linked to their professional development, access to dementia education, and organisational support.

### 4.1. Limitations

Only studies published in English were included. The search was limited to publications from 2009-2021. To ensure relevance to the UK care home sector, eligibility was limited to countries whose healthcare systems were similar to those of the UK. No study was excluded from the review based on quality. The papers included were of a variable standard which limits the interpretation and conclusions in this review.

## 5. Conclusions

Care home managers play an integral role in the delivery of person-centred care for people living with dementia in care homes. There is evidence of the characteristics of leadership that support the provision of person-centred dementia care within care homes. Less well understood is how leadership training interventions, and the hierarchy and organisation of the setting improve care home managers' leadership skills and competence to support staff to provide person-centred dementia care. Understanding what sustains and supports effective leadership over time to promote person-centred care for people living with dementia is less clear.

## 6. Implications for Practice

Care home managers who demonstrate that they value their staff and the involvement of residents and relatives in decision making can create a positive work environment and care culture. However, the care home environment can be challenging as staff shortages and time constraints limit care home managers' capacity to work closely with staff to promote person-centred approaches for care. Care home managers can be supported by their organisations and through training focused on developing their leadership in dementia care to acquire the skills to sustain quality care for people living with dementia.

## Figures and Tables

**Table 1:** Examples of electronic databases searched and terms used.

*PubMed*

(((((“leadership” [All Fields]) OR (“leader” [All Fields])) OR (“manager” [All Fields])) OR (“management” [All Fields])) AND ((((“care home” [All Fields]) OR (“residential home” [All Fields])) OR (residential care)) OR (“nursing home” [All Fields])) AND ((((((((“practice development” [All Fields]) OR (“action learning” [All Fields])) OR (education)) OR (training)) OR (staff support)) OR (“leadership support” [All Fields])) OR (“leadership skills” [All Fields]) OR person centred care OR quality of care OR culture change OR organizational culture OR organizational culture AND [Filter])))

*Cochrane library*

#1 MeSH descriptor: [Leadership] explode all trees #2 MeSH descriptor: [Residential facilities] explode all trees #3 MeSH descriptor: [Nursing homes] explode all trees

**Table 2:** An overview of twenty-one included studies' characteristics, primary focus and assessment tools and scores.

Study number	Author and year	Country	Design and data collection methods	Setting	Participants	Primary focus	Assessment tool and score
1	Backman et al. (2021)	Sweden	Quantitative cross-sectional study (questionnaires)	Nursing home	Managers/staff (*N* = 2429)	Leadership education and qualifications and person-centred care practices	AXIS
							Low

2	Cooke (2018)	Canada	Qualitative-focused ethnography study (observations and interviews)	Care home	Staff (*N* = 20)	Person-centred care practices	CASP
							Low

3	Dewar et al. (2019)	UK	Mixed methods (questionnaires and focus groups)	Nursing home	Managers (*N* = 119)	MHL leadership support programme	MMAT
							Low

4	Du Toit et al. (2020)	Australia	Qualitative study (delphi method/questionnaires)	Residential and nursing care home	Managers (*N* = 17) including: non-direct care staff	Person-centred care practices	CASP
							Low

5	Ericson-Lidman et al. (2014)	Sweden	Qualitative study (interviews)	Care home	Staff (*N* = 9)	Person-centred care practice	CASP
							Medium

6	Fossey et al. (2019)	UK	Qualitative study (focus groups)	Care home	Staff (*N* = 47)	WHELD programme	CASP
							Low

7	Havig et al. (2011)	Norway	Quantitative cross-sectional study (questionnaires)	Nursing home	Directors (*N* = 13) Managers (*N* = 40) Staff (*N* = 444) Relatives (*N* = 378)	Leadership style	AXIS
							Low

8	Hawkins et al. (2018)	UK	Qualitative ethnography study (observations and interviews)	Care home	Managers (*N* = 22) Relatives (*N* = 17) Staff (*N* = 444)	Staff training and supervision	CASP
							Medium

9	Hegelsen et al. (2014)	Norway	Qualitative study (interviews)	Nursing home	Staff (*N* = 11)	Dementia care training and education	CASP
							Medium

10	Kelley et al. (2020)	UK	Qualitative study (interviews)	Care home	Managers (*N* = 48)	DCM implementation	CASP
							Medium
	Griffiths et al. (2021)	UK	Qualitative study (interviews)	Care home	Managers (*N* = 2)	DCM implementation	CASP
					Staff (*N* = 18)		
							Low

11	O'Toole et al. (2021)	Australia	Qualitative study (interviews)	Care home	Managers (*N* = 18)	Leadership skills	CASP
							Low

12	Penney and Ryan (2018)	UK	Qualitative study (focus groups and interviews)	Care home	Managers (*N* = 15)	MHL leadership support programme	CASP
							Low

13	Poels et al. (2020)	USA	Quantitative cross-sectional study (questionnaires)	Nursing home	Directors (*N* = 9) Managers (*N* = 20) Staff (*N* = 235)	Leadership style	AXIS
							Medium

14	Quarsdorf & Bartholomeyczik (2019)	Germany	Qualitative case study (interviews)	Nursing home	Managers (*N* = 24)	DCM implementation	CASP
							Low

15	Ree (2020)	Norway	Quantitative cross-sectional study (questionnaires)	Nursing home and home care services	Staff (*N* = 165)	Leadership style	AXIS
							Low

16	Reon et al. (2018)	Norway	Quantitative cross-sectional study (questionnaires)	Nursing home and home care services	Staff (*N* = 1161)	Person-centred care practice	AXIS
							Low

17	Rokstad et al. (2015)	Norway	Qualitative study (focus groups and interviews)	Nursing home	Managers (*N* = 3)	DCM implementation	CASP
					Staff (*N* = 18)		Medium

18	Rutten et al. (2021)	Netherlands	Quantitative cross-sectional study (questionnaires)	Nursing home	Staff/nurses (*N* = 552)	Work environment, person-centred care and leadership style	AXIS
							Medium

19	Sjogren et al. (2015)	Sweden	Quantitative cross-sectional study (questionnaires)	Nursing home and care home	Staff (*N* = 1169)	Person-centred care practice	AXIS
							Medium

20	Smit et al. (2017)	Netherlands	Quantitative cross-sectional study (questionnaires)	Care home	Staff (*N* = 1389)	Leadership style	AXIS
					Residents (*N* = 1218)		Low

21	Surr et al. (2020)	UK	Qualitative collective case study (focus groups and interviews)	Care home	Managers and staff (no numbers given)	Dementia care training	CASP
							Medium

AXIS^1^ CASP^2^ DCM^3^ MMAT^4^ MHL^5^ WHELD^6^. ^1^Appraisal tool for cross-sectional studies. ^2^Critical appraisal skill programme. ^3^Dementia care mapping. ^4^Mixed methods appraisal tool. ^5^My Home Life. ^6^Well-being and health for people living with dementia.

**Table 3:** Contribution of included studies to three main themes.

Authors	Leadership performance and approach	Importance of dementia training and education	Characteristics of effective and ineffective leadership and management
Backman et al. (2021)	X		X
Cooke (2018)	X		X
Dewar et al. (2019)	X		X
Du Toit et al. (2020)	X	X	
Ericson-Lidman et al. (2014)	X	X	
Fossey et al. (2019)	X	X	
Griffiths et al. (2021)	X	X	
Havig et al. (2011)			X
Hawkins et al. (2018)		X	X
Hegelsen et al. (2014)	X	X	
Kelley et al. (2020)	X	X	X
O'Toole et al. (2020)	X		X
Penney & Ryan (2018)	X		
Poels et al. (2020)			X
Quarsdorf & Bartholomeyczik (2019)	X	X	X
Ree (2020)			X
Reon et al. (2018)	X		X
Rokstad et al. (2015)	X	X	X
Rutten et al. (2021)			X
Sjogren et al. (2015)	X	X	X
Smit et al. (2017)		X	X
Surr et al. (2019)	X	X	X

## Data Availability

The data that support the findings of this study are available from the corresponding author upon reasonable request.

## Supplementary Materials

Supplementary Materials Supporting Information 1: Appraisal tools and scoring criteria used to assess studies for risk of bias.
