# Supplementary material for: The Influence of Care Home Managers' Leadership on the Delivery of Person-Centred Care for People Living with Dementia: A Systematic Review
Source: J Nurs Manag. 2023 May 17;2023:9872272. doi: 10.1155/2023/9872272 (PMC11918814; doi:10.1155/2023/9872272)
Supplement: Supplementary Materials — Supporting Information 1: Appraisal tools and scoring criteria used to assess studies for risk of bias. [file 9872272.f1.docx]

**Supporting Information 1**

*Appraisal Tools and Scoring Criteria Used to Assess Studies for Risk of Bias*

| Appraisal Tool | Scoring Criteria |
| --- | --- |
| Appraisal tool for Cross-sectional Studies [AXIS] (Downes et al., 2016)  Scored as yes, no, don’t know | **1** Were the aims/objectives of the study clear?  **2** Was the study design appropriate for the stated  aim(s)?  **3** Was the sample size justified?  **4** Was the target/reference population clearly defined?  (Is it clear who the research was about?)  **5** Was the sample frame taken from an appropriate  population base so that it closely represented the  target/reference population under investigation?  **6** Was the selection process likely to select  subjects/participants that were representative of the  target/reference population under investigation?  **7** Were measures undertaken to address and  categorise non-responders?  **8** Were the risk factor and outcome variables  measured appropriate to the aims of the study?  **9** Were the risk factor and outcome variables  measured correctly using instruments/measurements  that had been trialled, piloted or published previously?  **10** Is it clear what was used to determined statistical  significance and/or precision estimates? (e.g. p-  values, confidence intervals)  **11** Were the methods (including statistical methods)  sufficiently described to enable them to be repeated?  **12** Were the basic data adequately described?  **13** Does the response rate raise concerns about non-  response bias?  **14** If appropriate, was information about non-responders  described?  **15** Were the results internally consistent?  **16** Were the results presented for all the analyses  described in the methods?  **17** Were the authors' discussions and conclusions  justified by the results?  **18** Were the limitations of the study discussed?  **19** Were there any funding sources or conflicts of  interest that may affect the authors’ interpretation of  the results?  **20** Was ethical approval or consent of participants  attained? |
| Critical Appraisal Skill Programme [CASP] (Long et al., 2020)  Scored as yes, no, can’t tell | **1** Was there a clear statement of the aims of the  research?  **2** Is a qualitative methodology appropriate?  **3** Was the research design appropriate to address the  aims of the research?  **4** Was the recruitment strategy appropriate to the aims  of the research?  **5** Was the data collected in a way that addressed the  research issue?  **6** Has the relationship between researcher and  participants been adequately considered?  **7** Have ethical issues been taken into consideration?  **8** Was the data analysis sufficiently rigorous?  **9** Is there a clear statement of findings?  **10** How valuable is the research? |
| Mixed Methods Appraisal Tool [MMAT] (Hong et al., 2018)  Scored as yes, no, can’t tell | **1** Is the qualitative approach appropriate to answer the  research question?  **2** Are the qualitative data collection methods adequate to  address the research question?  **3** Are the findings adequately derived from the data?  **4** Is the interpretation of results sufficiently substantiated  by data?  **5** Is there coherence between qualitative data sources,  collection, analysis and interpretation?  **6** Is randomisation appropriately performed?  **7** Are the groups comparable at baseline?  **8** Are there complete outcome data?  **9** Are outcome assessors blinded to the intervention  provided?  **10** Did the participants adhere to the assigned  intervention?  **11** Are the participants representative of the target  population?  **12** Are measurements appropriate regarding both the  outcome and intervention (or exposure)?  **13** Are there complete outcome data?  **14** Are the confounders accounted for in the design and  analysis?  **15** During the study period, is the intervention  administered (or exposure occurred) as intended?  **16** Is the sampling strategy relevant to address the  research question?  **17** Is the sample representative of the target population?  **18** Are the measurements appropriate?  **19** Is the risk of nonresponse bias low?  **20** Is the statistical analysis appropriate to answer the  research question?  **21** Is there an adequate rationale for using a mixed  methods design to address the research question?  **22** Are the different components of the study effectively  integrated to answer the research question?  **23** Are the outputs of the integration of qualitative and  quantitative components adequately interpreted?  **24** Are divergences and inconsistencies between  quantitative and qualitative results adequately  addressed?  **25** Do the different components of the study adhere to  the quality criteria of each tradition of the methods  involved? |
